# The Interventricular Septum: Structure, Function, Dysfunction, and Diseases

**DOI:** 10.3390/jcm11113227

**Published:** 2022-06-06

**Authors:** Filippos Triposkiadis, Andrew Xanthopoulos, Konstantinos Dean Boudoulas, Grigorios Giamouzis, Harisios Boudoulas, John Skoularigis

**Affiliations:** 1 Department of Cardiology, University Hospital of Larissa, 41110 Larissa, Greece; andrewvxanth@gmail.com (A.X.); grgiamouzis@gmail.com (G.G.); iskoular@gmail.com (J.S.); 2 Division of Cardiovascular Medicine, The Ohio State University, Columbus, OH 43210, USA; konstantinos.boudoulas@osumc.edu; 3 Biomedical Research Foundation, Academy of Athens, 11527 Athens, Greece; boudoulas@bioacademy.gr

**Keywords:** interventricular septum, structure, function, diseases, treatment

## Abstract

Vertebrates developed pulmonary circulation and septated the heart into venous and arterial compartments, as the adaptation from aquatic to terrestrial life requires more oxygen and energy. The interventricular septum (IVS) accommodates the ventricular portion of the conduction system and contributes to the mechanical function of both ventricles. Conditions or diseases that affect IVS structure and function (e.g., hypertrophy, defects, other) may lead to ventricular pump failure and/or ventricular arrhythmias with grave consequences. IVS structure and function can be evaluated today using current imaging techniques. Effective therapies can be provided in most cases, although definitions of underlying etiologies may not always be easy, particularly in the elderly due to overlap between genetic and acquired causes of IVS hypertrophy, the most common being IVS abnormality. In this review, state-of-the-art information regarding IVS morphology, physiology, physiopathology, and disease is presented.

## 1. Introduction

Vertebrates changed the structure and function of their hearts in order to adapt to the living environment over the course of evolution. Since adaptation from aquatic to terrestrial life requires more oxygen and energy, vertebrates developed a pulmonary circulation and septated the heart into venous and arterial compartments, allowing the supply of oxygenated blood to peripheral tissues [1]. The formation of the interventricular septum (IVS) initiates at around the fifth week of embryonic development; it involves the sequential fusion of three independent septa: muscular, outlet, and inlet [2]. Cardiac ventricular septation, besides its great intrinsic interest to evolutionary biologists, is also crucial to the physiologist and the clinician. IVS function is important in the healthy heart. IVS contributes to normal left and right ventricular (LV and RV respectively) function, not only via its position and movement, but also through the regulation of RV and LV interaction (ventricular interdependence) [3]. On the other hand, the IVS may become abnormal either from disease of the IVS itself (congenital anomalies, coronary artery disease, cardiomyopathy, hypertension, conduction abnormalities, tumors, etc.) or as a result of abnormalities that involve other structures of the heart that induce abnormal hemodynamics in the IVS (atrial septal defect, valvular heart disease, open heart surgery, etc.) [4].

In this review, an update on adult IVS morphology, physiology, physiopathology, and disease is provided.

## 2. IVS Structure

Traditionally, gross cardiac anatomy has been described mainly based on the findings in the dissection suite. However, imaging with echocardiography, cardiac computed tomography (CCT), and cardiac magnetic resonance (CMR), have fundamentally advanced the understanding of cardiac anatomy in the 21st century [5]. Additionally, CMR is unique for its tissue characterization capabilities, and for the identification of myocardial scar, fibrosis, edema, and inflammation. Diffusion tensor cardiovascular magnetic resonance (DT-CMR) is a noninvasive technique that has been increasingly used to unravel human cardiovascular microstructure in vivo. Finally, it is noteworthy that the IVS thickness prenatally assessed using ultrasound imaging may be a useful diagnostic marker of fetal macrosomia, a common condition affecting approximately 10% of fetuses, and potentially leading to serious complications in both mother and child [6].

The IVS is composed of a muscular and a membranous component. The muscular IVS component arises from the endocardial surface of the common ventricle, and has a normal thickness of approximately 9 mm in women and 10 mm in men [7]. The comparatively diminutive membranous component, the fibrous component of the IVS, is found between the outflow tracts of the LV and RV [8]. Animal studies that examined the transmural features of the IVS reported that the myofiber angle shifted from a longitudinal orientation on the LV-side to a circumferential orientation in the midwall, and then back to a longitudinal orientation on the RV-side of the septum. The main transmural change in the septum was related to the orientation of the myofibers (anisotropy), but not to the mechanical properties or tissue composition [9].

Blood supply to the IVS is predominantly derived from penetrating arteries entering from the left anterior descending artery, whereas branches from the posterior descending artery supply only the posterior third of the IVS [10]. The IVS is a site of collateral circulatory channels in the human heart. The middle cardiac vein, which empties into the coronary sinus, is responsible for draining the IVS [11].

Cardiac function is controlled by the autonomic nervous system, which consists of the parasympathetic and sympathetic divisions. In addition to the complex integration that occurs in the central nervous system, there is local intracardiac neural regulation brought about by a network of ganglionated plexuses consisting of a large number of intracardiac ganglia, and gathered in specific heart regions including the atria, the sinoatrial and atrioventricular nodes, the IVS, and both ventricles [12].

The IVS accommodates important parts of the cardiac conduction system. The atrioventricular node is located at the apex of the Koch triangle, whereas the bundle of His divides at the juncture of the fibrous and muscular boundaries of the IVS into the left and right bundle branches (LBB and RBB, respectively). The LBB subsequently divides into anterior and posterior fascicles, which course towards the anterior and posterior papillary muscles of the LV, whereas the RBB courses down the right side of IVS near the endocardium in its upper third, deeper in the muscular portion of the IVS in the middle third, and then again near the endocardium in its lower third [13].

Both ventricles contribute to IVS formation [14]. Cardiomyocyte helical architecture is present as early as the midgestational period [15], whereas cardiomyocyte transformation affecting the IVS initiates in the early postnatal period, whereby the RV origin of the IVS is reduced from 50% in the perinatal period to 25% later in life [15]. A highly echogenic zone seen in the IVS during cardiac ultrasound coursing from base to apex corresponds to the position of the circumferentially orientated cardiomyocytes.

## 3. IVS Function

During systole the IVS thickens and moves towards the LV after the onset of electrical depolarization followed by a brief ‘shudder’ at end systole, whereas during diastole it returns to its original thickness and position [16]. The IVS sides are significantly softer than their corresponding ventricular free walls, and the collagen content is less than that of the ventricular walls [9]. Moreover, at low strains, there is similar anisotropic behavior between the two sides, whereas at high strains, both sides are isotropic.

Normal septal position and twisting are essential for ventricular function, whereas the functional interaction between the RV and LV via IVS has been termed ‘‘ventricular interdependence” [3,17].

### 3.1. Ventricular Interdependence

Ventricular interdependence describes the influence that each ventricle has to the other as a result of the shared IVS [18], the encircling of the two ventricles by common myocardial fibers, and the pericardial constrain [19]. An intact pericardium enhances ventricular diastolic interdependence but has negligible effect on ventricular systolic interdependence, which is affected by IVS and free wall properties [20]. The RV contributes significantly to the normal cardiac output response to exercise, as demonstrated by the 30–40% decrease in maximum oxygen uptake in young patients with Fontan physiology compared with healthy controls [21]. Ventricular interdependence has also been implicated in the development of RV remodeling in untreated asymptomatic mild hypertensive patients, and has been attributed to changes in IVS structure [22].

### 3.2. Abnormal IVS Motion

Abnormal, paradoxical septal motion (PSM), is a type of motion abnormality where the IVS movement is atypical for the particular phase of the cardiac cycle.

The most common cause of PSM is left bundle branch block (LBBB) due to periods of asynchrony in contraction, ejection, end-systole, and end-diastole between RV and LV, in addition to decreased regional ejection fraction (EF) of the IVS. The LBBB usually results in two types of PSM, namely septal flash and apical rocking, which are interrelated [23,24]. Septal flash refers to a leftward motion of the IVS associated with a marked pre-ejection shortening; this is mainly due to active IVS contraction, which is followed by immediate re-lengthening (rebound stretch) resulting from contractions in late-activated remote myocardium and subsequent paradoxical rightward IVS motion [25]. Apical rocking, in turn, is characterized by a short septal motion of the apex caused by the contraction of the IVS early in systole, and a subsequent long motion to the lateral side during ejection resulting from the late lateral contraction caused by the LBBB [23].

IVS ischemia/infarction is another common cause of abnormal septal motion. Experi-mental studies have demonstrated that following septal artery ligation, IVS shortening is immediately replaced by systolic lengthening [26]; eventually, IVS hypokinesia or akinesia develop. The cardiogenic shock, which is frequently due to acute myocardial infarction (AMI), depends on AMI extent and its complications, the most important being mitral regurgitation, IVS rupture, and rupture of the LV free wall [27].

Diastolic and systolic ventricular interactions are negatively affected in pulmonary arterial hypertension (PAH) and result in PSM. In the early stages of PAH, a rapid leftward IVS motion occurs during early LV diastole which is most likely due to prolonged RV myocardial activation [28]. Severe PAH results in RV failure with RV eccentric remodeling and contractile dysfunction, and has an important impact on the geometry, structure, and function of the LV. RV volume and pressure increases cause mechanical IVS flattening and shifting towards the LV, leading to LV compression visualized by paradoxical IVS motion, a “D-shaped” LV, and an increased LV eccentricity index [29]. RV systolic and biventricular diastolic dysfunction result in a reduction of cardiac output and coronary blood flow, both of which may exacerbate congestion [30].

A common cause of PSM is constrictive pericarditis, which is characterized by dissociation of intrathoracic from intracardiac diastolic pressures; it exaggerates diastolic ventricular interdependence due to the fixed volume in the pericardial sack resulting from the thickened, fibrotic, and/or calcified non-compliant pericardium [31]. With inspiration, the RV cannot expand to accommodate increased venous return and encroaches into the LV space, via a shift of the IVS; this results in decreased LV filling and output. The preferential filling of the right heart chambers during inspiration subsequently gives way to preferential left heart filling with expiration, when increased intrathoracic pressure decreases systemic venous return to the right heart, restores the filling gradient between the pulmonary veins and left heart chambers, and shifts the IVS towards the RV [32].

PSM may also occur after uncomplicated cardiac surgery resulting from an increase in RV transverse shortening (free wall to septal fibers), in order to compensate for the reduced RV longitudinal shortening [33], atrial septal defect (ASD) to accommodate the increased RV volume [34], and mitral stenosis reflecting an abnormal transseptal gradient [35].

## 4. Disorders and Diseases Affecting the IVS

### 4.1. Hypertrophic Cardiomyopathy

HCM is characterized by increased LV wall thickness in the absence of abnormal loading conditions, such as arterial hypertension or aortic valve stenosis, that can stimulate this magnitude of IVS hypertrophy. HCM is a common inherited cardiovascular disease with a prevalence of one in 200–500 adults in the general population [36].

HCM has been increasingly recognized as having a complex genetic etiology [37]. Most HCM patients remain asymptomatic or mildly symptomatic throughout life, whereas others have dyspnea, exercise intolerance, chest pain, palpitations, presyncope, syncope, and sudden cardiac death (SCD). SCD may be the initial presentation [38].

**Figure 1:**
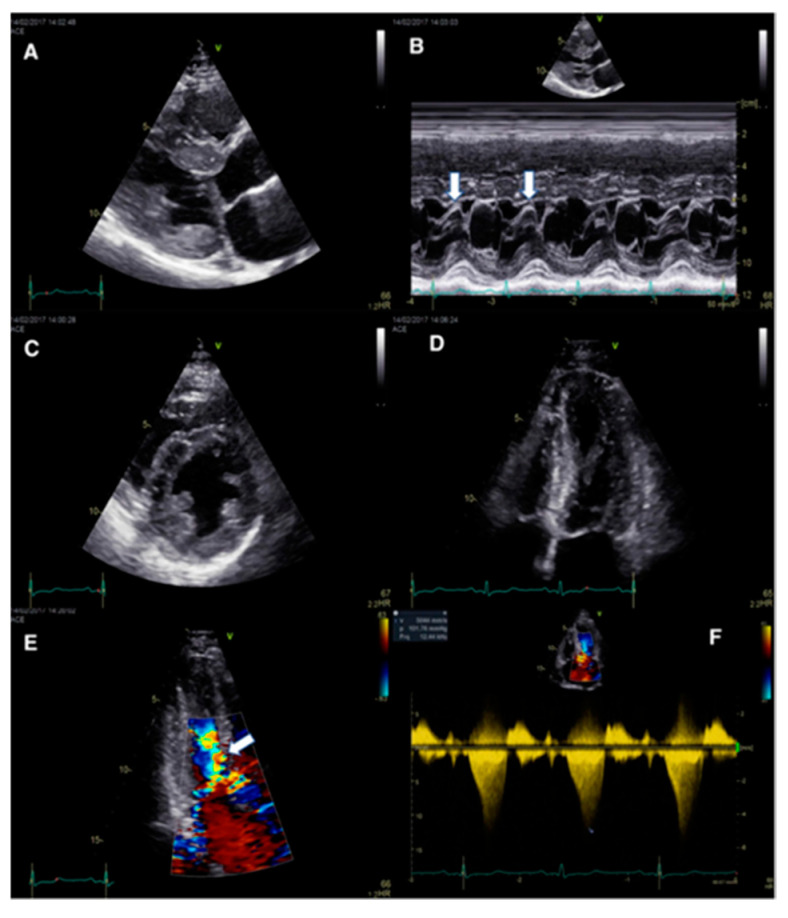
Complex mechanisms leading to dynamic obstruction in a patient with HCM. Concentric hypertrophy involving mainly the basal septum (diastolic IVS thickness of 15 mm), and elongated mitral leaflets with systolic anterior motion (**A**); M-mode echocardiography shows the systolic contact of the mitral valve with the IVS (arrows) (**B**); anterior displacement of the hypertrophied papillary muscles (**C**,**D**); moderate eccentric (posteriorly oriented) mitral regurgitation secondary to SAM (**E**); and significant resting LVOT obstruction by CW Doppler (peak resting gradient of 102 mmHg) (**F**). Of note, there is severe LVOT obstruction without severe septal hypertrophy, explained by the significant abnormalities of the mitral valve apparatus. HCM hypertrophic cardiomyopathy, IVS interventricular septum, LVOT left ventricular outflow tract. Mandeş L, et al. Journal of Echocardiography 2020; 18: 137–148 [39].

HCM diagnosis is confirmed usually with echocardiography or CMR (Figure 1) [39]. Any segment of the LV can be involved, although HCM is classically asymmetric and mainly involves the IVS. RV dysfunction is often observed in HCM. The main histological features of HCM are cardiomyocyte disarray and fibrosis [40]. Mutations in cardiac β-myosin account for 35% of genetically based HCM. The majority of variants causing HCM increase the proportion of active myosin which consequently leads to amplified force production in systole (hypercontractility) and diastole (diastolic dysfunction) [41]. It has been proposed that the molecular basis of this hypercontractility in HCM can be described by three perspectives: (a) changes in the fundamental parameters of the actin-activated β-cardiac myosin chemo-mechanical ATPase cycle; (b) an increase in the number of functionally accessible heads in the sarcomere for interaction with actin; and (c) HCM mutations and small-molecule effectors leading to changes in load dependence and power output of cardiac contraction [42]. It is noteworthy that a recently discovered energy conserving state of myosin, the super relaxed state, is pivotal to modulating force production and energy use within the sarcomere [43]; this can be disrupted by HCM cardiac myosin mutations [44]. One of the most well recognized functional abnormalities in HCM is the dynamic LV outflow tract (LVOT) obstruction occurring in 60–70% of patients at rest, or with physiologic provocation [45]. “Athlete’s Heart” may overlap with some features of HCM, and the distinction between physiological versus pathological changes in athletes is imperative [46].

### 4.2. Basal Septal Hypertrophy (Upper Septal Hypertrophy or ‘Sigmoid Septum’)

**Figure 2:**
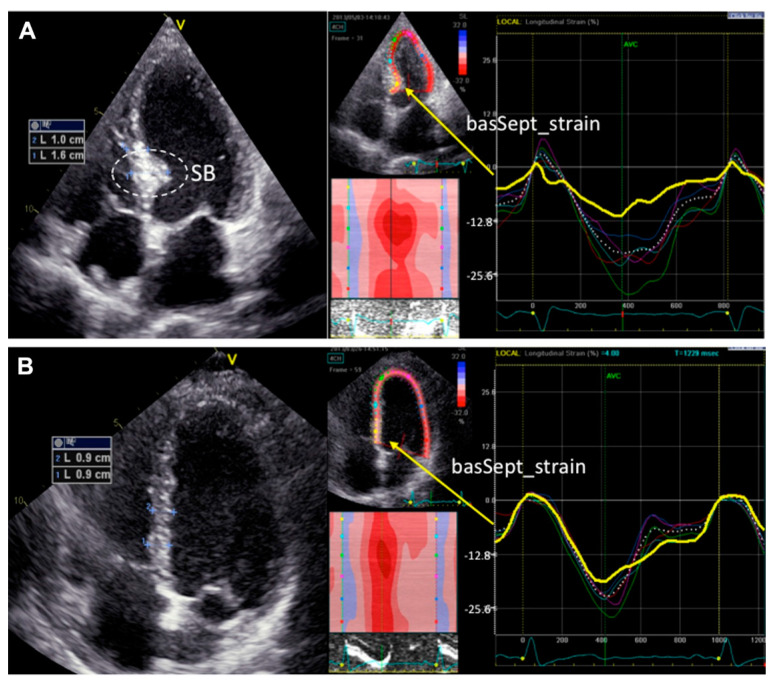
Examples of segmental longitudinal systolic strain curves derived from speckle tracing imaging in patients with (**A**) or without (**B**) septal bulge (SB). Gaudron PD, et al. J Am Soc Hypertens 2016; 10: 70–80 [49].

Basal septal hypertrophy (BSH) may be present in approximately 20–25% of patients with hypertension [47] or aortic stenosis [48], and is associated with a decrease in the regional myocardial deformation of the basal-IVS (Figure 2) [49]. In some patients, BSH may increase the risk of dynamic LVOT obstruction even in the absence of underlying hypertrophic obstructive cardiomyopathy [50]. Based on the above, BSH has been proposed as an early imaging biomarker of risk for the progression of hypertensive disease to heart failure [51]. However, the precise definition of the underlying BSH etiology is not always easy, especially in the elderly, where there is overlap between genetic and acquired causes of IVS hypertrophy [52].

### 4.3. Ventricular Septal Defects

Ventricular septal defect (VSD) accounts for 10% of congenital heart defects in adults [53]. VSD in the adult can also occur as an acquired condition, such as a complication after acute myocardial infarction (MI) (see below), surgical or transcatheter aortic valve replacement [54], septal myectomy for HCM [55], erosion of a strut of a bioprosthetic mitral valve [56], or from stress (Takotsubo) cardiomyopathy [57]. VSDs vary in size, ranging from small defects without hemodynamical significance, to large communications leading to complications in early childhood [58]. A decrease in shunt size or even spontaneous closure of VSDs is common during early childhood. Depending on size and physiology, clinical presentation and findings vary considerably. Small defects typically cause a loud pansystolic murmur often associated with a palpable thrill, whereas in large defects with ventricular pressure equalization, no murmur is audible. Patients with elevated pulmonary vascular resistance and shunt reversal show signs of central cyanosis with clubbing of hands and feet.

### 4.4. Septal Infarction

Isolated septal MI is uncommon and may be due to occlusion of the septal perforator branches that supply the anterior portion of the IVS and the bundle of His. Septal involvement in MI is usually due to occlusions of the LAD or dominant right coronary or dominant left-circumflex arteries, since all of these give rise to septal branches and may lead to IVS rupture [59]. Occasionally, IVS rupture may occur, establishing a communication between the two ventricles. In the era of early reperfusion therapy, however, post-MI VSD is a rare (<1% of MI patients) but potentially catastrophic complication [60]. Post-MI IVS rupture and VSD occur 3 to 8 days after a transmural acute MI.

### 4.5. Ventricular Arrhythmias and Conduction Abnormalities

The IVS is important as part of the ventricular tachycardia (VT) substrate in several conditions such as idiopathic VT [61] as well as in VT, as a result of ischemic heart disease (IHD) [62], HCM [63] and dilated cardiomyopathy (DCM) [64]. Further, patients with ischemic heart disease may develop heart block due to ischemia or infarction of the atrioventricular conducting pathway. The atrioventricular node artery is a major contributor to the arterial supply of the atrioventricular conducting pathway, and is an important vessel in the pathogenesis of heart block. It is noteworthy that in a study in which the terminal ramifications of this artery were evaluated by serial sectioning in 50 human hearts, the atrioventricular artery provided branches to the posterior IVS in all hearts (100%) and to the interatrial septum in 22 hearts (44%). The vessel supplied the atrioventricular node in 45 hearts (90%), whereas it supplied the penetrating bundle in 32 hearts (64%) [65].

### 4.6. Other

**Figure 3:**
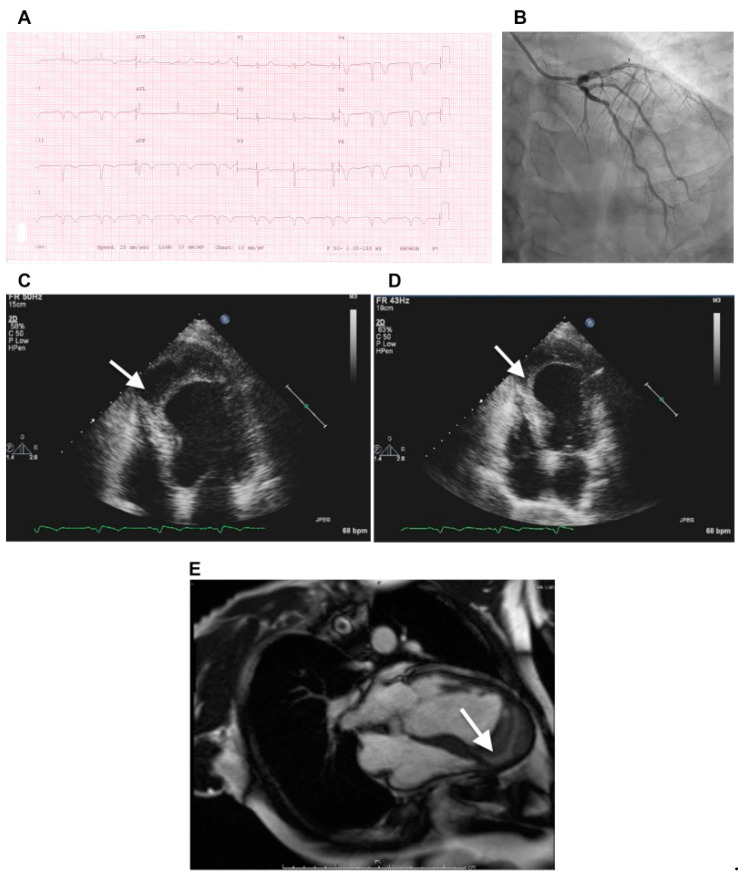
(**A**) Electrocardiogram at presentation shows biphasic T wave in lead V3, deep T-wave inversion in leads V4, V5, and V6, and Q waves with T-wave inversion in the inferior leads, consistent with angiographic findings. (**B**) Coronary angiogram shows moderate stenosis at the proximal segment of the left anterior descending coronary artery, severe stenosis at midsegment, and complete total occlusion in the distal segment. (**C**–**E**) Transthoracic two-dimensional echocardiographic and cardiac magnetic resonance views of IDH. (**C**,**D**) At presentation, apical four-chamber view showing dissecting echo-free cavity (arrow). (**E**) An IDH was confirmed by gadolinium-enhanced magnetic resonance imaging, revealing a large thrombus (arrow) within the apical intramyocardial dissection cavity containing the hematoma in the apical segment. Roslan A, et al. CASE (Phila). 2017 August; 1(4): 159–162 [69].

Less common pathologies affecting the IVS include aneurysm [66], diverticulum [67], and lipomatous hypertrophy [68]. Intramyocardial dissecting hematoma is a rare form of cardiac rupture that may occur following MI, chest trauma, or percutaneous intervention. It can develop in the LV free wall, RV, or IVS, and consists of blood infiltration into and through the myocardial wall (Figure 3) [69].

**Figure 4:**
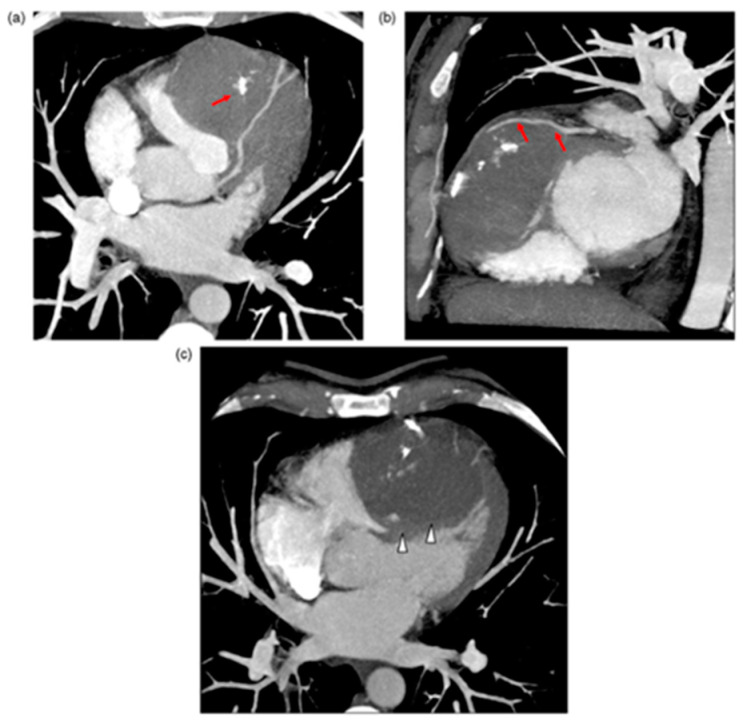
(**a**) Multi-detector computed tomography (MDCT) axial transverse view. Right ventricular (RV) mass occupying the ventricle and bulging out. The mass is pushing out the RV free wall. Note calcifications in the mass (red arrows). (**b**) MDCT right oblique short axis view. Left anterior descending artery in contact with fibrous mass tissue (arrows). (**c**) MDCT with multiplanar reconstruction and Maximum Intensity Pixel on four chamber view. Absence of pericardial effusion. The mass is arising from the IVS (white arrowheads); large sessile adherence on IVS. Stéphant E, et al. Eur. J. Radiol. Extra 2008; 67: e103–e106 [72].

IVS abscess development usually occurs as an extension of infective endocarditis from cardiac valves and is associated with high mortality [70]. Cardiac tumours are some of the rarest primary tumours, while cardiac metastases are more common yet still relatively rare [71]. Seventy-five percent of primary cardiac tumours are benign and seldom involve the IVS. Fibromas and hemangiomas occasionally originate in the IVS and may mimic HCM (Figure 4) [72], whereas hamartomas of mature cardiac myocytes (HMCM) are hyperproliferative growth of mature cardiac cells, which are slow growing and solitary, usually present in young men in their mid-twenties in the ventricles and IVS; they may be asymptomatic.

**Figure 5:**
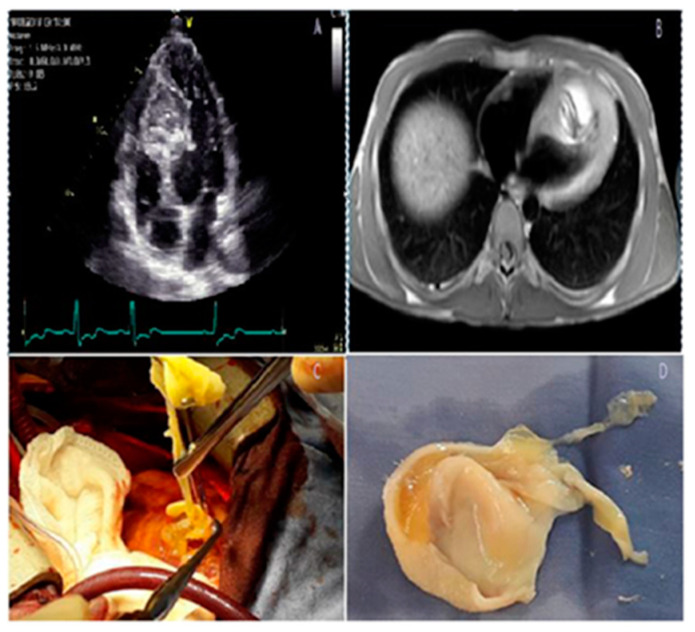
A large cardiac hydatid cyst in the interventricular septum. (**A**) Echocardiographic frame in an apical 4-chamber view showing a large cystic mass splitting the interventricular septum (IVS). (**B**) Magnetic resonance image showing hydatid cyst located in the IVS. (**C**) Cystectomy and membrane extraction. (**D**) Germinative membrane. Fennira S, et al. Int. J. Infect Dis. 2019;78:31–33 [74].

Cardiac thrombi may occur in 2–7% in patients with atrial fibrillation or LV dysfunction, with thrombus formation occurring at the IVS in approximately 11% of the cases [73]. IVS rupture as a mechanical complication of MI may lead to thrombus formation. Cardiac involvement including the IVS is rare in cystic hydatid disease (CYHD) [74]. Echocardiography is useful for the diagnosis, whereas CCT and CMR provide further information, such as the extent and anatomic relationships of the cysts (Figure 5) [74].

## 5. Treatment Modalities Targeting Diseases of the IVS

### 5.1. Cardiac Resynchronization Therapy (CRT)

CRT is an established treatment in selected patients with heart failure [75]. CRT in most cases is achieved by adding a LV pacing lead (lateral or posterolateral wall of the LV) to a standard pacemaker or defibrillator system that generally includes only an RV lead (RV apex) and—in cases of sinus rhythm—a right atrial lead [76]. The mechanisms of action of CRT in this setting include correction of IVS abnormal systolic motion and restoration of LV electrical and mechanical synchrony [77]. Circumferential contraction of IVS myocardial fibers is improved with CRT, and it is strongly correlated with an increase in aortic velocity time integral (VTI) and shortening of QRS duration [78]. Therefore, not surprisingly, CRT improves cardiac function and symptoms, as well as reduces morbidity and mortality in a specific group of heart failure patients with abnormal LVEF and QRS duration. Nevertheless, approximately 20–40% of the abovementioned patients may not respond to CRT (non-responders) [79].

### 5.2. Mavacamten

Mavacamten is a small molecule that belongs to the myosin modulators, a novel class of pharmaceutical agents that are being developed to treat patients with a range of cardio-myopathies. Myosin modulators are often classified as either “myosin activators” (omecamtiv, danicamtiv) or “myosin inhibitors” (mavacamten, aficamten) [80]. The therapeutic goal of these drugs is to target cardiac myosins directly to modulate contractility and cardiac power output, in order to alleviate symptoms that lead to heart failure and arrhythmias without changes in calcium signaling [81].

In the recently completed EXPLORER-HCM (Clinical Study to Evaluate Mavacamten [MYK-461] in Adults with Symptomatic Obstructive Hypertrophic Cardiomyopathy), about one-third of patients on mavacamten, a myosin inhibitor that reduces myocardial contractility, achieved the primary end-point of subjective symptomatic improvement and increased functional capacity assessed by peak VO2 [82]. This beneficial effect has been attributed to mavacamten-induced decrease in LVOT gradients and resolution of mitral valve systolic anterior motion in most HCM patients [83].

### 5.3. Septal Reduction Therapy (SRT)

SRT reduces LVOT obstruction in patients with HCM and includes surgical IVS myectomy and transcoronary alcohol IVS ablation. The role of surgical IVS myectomy in HCM is well established. Transcoronary alcohol IVS ablation provides a less invasive approach to septal reduction in HCM. Both surgical IVS myectomy and transcoronary alcohol IVS ablation may improve HCM patients’ functional status, with low periprocedural mortality and excellent long-term survival [84]. As the results of these two treatment options seem to be comparable in experienced centers, selection depends on the anatomical findings, concomitant cardiac and non-cardiac morbidities, technical issues, the operator’s expertise and availability, and patient’s choice [85].

### 5.4. VSD Closure

VSD is the most common congenital heart defect. VSD creates a shunt between the RV and LV. Untreated medium or large VSDs lead to congestive heart failure [86]. Surgical closure remains the treatment of choice for most defects. However, transcatheter closure of muscular VSD has emerged as a safe and effective alternative [87]. Moreover, it has been recently demonstrated that transcatheter device closure of perimembranous VSD and intracristal VSD can be performed safely and successfully in selected patients with excellent medium- and long-term results [88]. Interestingly, repair of post-MI septal rupture with transcatheter defect closure emerges as a viable option [89].

### 5.5. IVS Ablation of VT

Ventricular arrhythmias originating from the LV summit and IVS account for 10–15% of ventricular arrhythmias. Managing those arrhythmias is a major challenge. Novel mapping and ablation strategies are needed in order to treat arrhythmias originating from these regions, given the current suboptimal long-term success rates with standard techniques [90]. In this regard, bipolar radiofrequency ablation has emerged as a promising technique for the treatment of septal VTs in patients with non-ischemic dilated cardiomyopathy [91]. However, it was recently reported that idiopathic ventricular fibrillation (IVF) in which a triggering premature ventricular complexes leading to the IVF episodes could not be identified, was successfully treated using posterior fascicle transection and empirical linear ablation of the mid-Purkinje potentials identified along the LV interventricular inferior septum [92].

## 6. Conclusions

**Figure 6:**
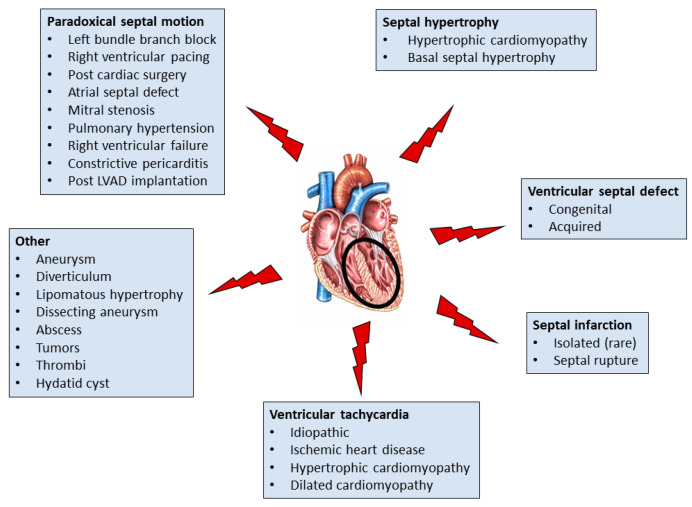
Conditions and disorders affecting the interventricular septum in the adult. LVAD, left ventricular assist device.

The IVS plays a pivotal role in cardiac function as it contributes to the structure and function of both ventricles. Conditions or diseases that affect the IVS may lead to pump failure and/or ventricular arrhythmias with grave consequences (Figure 6). A detailed evaluation of the IVS is feasible with current noninvasive imaging modalities, and effective therapy can be implemented in most cases with IVS involvement. Definition of the underlying etiology may not always be easy, especially in the elderly there is overlap between genetic and acquired causes of IVS pathology, particularly in IVS hypertrophy, the most common abnormality. The rapid developments in medical science and technology are anticipated to help better define the mechanisms underlying IVS pathology and lead to accurate diagnoses, better management, and hopefully prevention at least in certain cases.

## Data Availability

Not applicable.
